# Comparative Study of Sampling and Measurement Methods for the Development of CH_4_ Emission Factors at MSW Incinerators

**DOI:** 10.3390/ijerph19148647

**Published:** 2022-07-15

**Authors:** Seongmin Kang, Jiyun Woo, Eui-chan Jeon

**Affiliations:** 1 Climate Change & Environment Research Center, Sejong University, Seoul 05006, Korea; smkang9804@gmail.com; 2 Department of Climate and Environment, Sejong University, Seoul 05006, Korea; woojune92@gmail.com

**Keywords:** climate change, greenhouse gas, waste incineration, measurement method

## Abstract

Lung samplers (periodic sampling) have generally been used to develop CH_4_ emission factors in waste incineration facilities. Since this method must be calculated using only the value at a specific point in time, it may not reflect the emission characteristics depending on the circumstances of the facility. In order to supplement this method, a method of continuously collecting samples for a long period of time or continuously measuring may be used. In this study, the CH_4_ emission factor development and titration methodology were reviewed using both the existing methods and the newly proposed continuous sampling and continuous measurement methods. As a result of the analysis, the average emission factor by periodic sampling was 0.201 gCH_4_/ton-waste, the average emission factor by continuous capture was 0.199 gCH_4_/ton-waste, and the average emission factor by continuous measurement was 0.176 gCH_4_/ton-waste. There was a difference of 0.025 gCH_4_/ton-waste in the emission factor values by periodic sampling and continuous measurement, and the emission factor values for periodic sampling and continuous sampling were similar. There was no statistically significant difference, confirming that all three methods could be used. However, the existing method, periodic sampling, cannot reflect the characteristics of the night, and, in the case of continuous measurement, expensive equipment and maintenance are difficult. Therefore, it is judged that the method using continuous sampling is a good method that can combine the two advantages.

## 1. Introduction

Waste generated by human activities and its treatment is causing several environmental problems. Recycling is the most common waste treatment method among others, such as landfills and incineration, used in South Korea [1]. The amount of landfill treatment was higher than that of incineration until 2019; however, the quantity of incinerated waste was 1015 million tons/year in 2020, approximately 13 million tons greater than that of landfill waste of 1002 million tons/year [2]. As major sources of all greenhouse gas emissions (approximately 83%) in the waste treatment sector, landfills and incineration require careful management. With the increasing amount of incineration, relevant studies on greenhouse gas emissions and management have become even more necessary [3,4,5,6].

An accurate estimation of greenhouse gas emissions must precede any effective response to climate change. Owing to the reduced priority of CH_4_ and N_2_O because of relatively lower emissions compared with CO_2_, which accounts for most of the greenhouse gases, the requirement of relevant studies is even greater, and it is important to acquire emission factors for estimating emissions based on emission concentration measurements [7,8,9]. In the case of waste incineration, CO_2_ emissions are currently calculated using the analyzed value of each waste characteristic, whereas the emission factors and amounts for CH_4_ and N_2_O are calculated using gas measurements [10,11,12]. As the calculated CH_4_ and N_2_O emissions may be significantly affected by the method of measurement, an adequate methodology should be considered to develop representative emission factors. In addition, accurate calculation of emissions is very important for managing emissions of waste incineration facilities, which may affect the health of residents living in the surrounding area [13,14].

Lung samplers are generally used to develop CH_4_ emission factors in waste incineration facilities, and many related studies have been conducted [15,16,17,18,19]. However, collecting samples at night with this method is difficult owing to safety-related issues in the facilities, and the nighttime emission characteristics are not reflected. While the periodic collection method allows good portability, obtaining a representative concentration measurement of the entire section is limited because samples are collected only at specific time points.

To supplement this method, it is possible to consider a method of collecting samples for a long period of time for analysis or collecting data in real time using a continuous measuring instrument. Therefore, this study attempts to compare the values after calculating the CH_4_ emission factor using the method using a lung sampler, which is the most frequently used method (periodic sampling), continuously collecting samples (continuous sampling), and the real-time concentration measurement method (continuous measurement). Through a comparison of these methodologies, we intend to find a way to increase the reliability of the CH_4_ emission factor development in waste incineration facilities.

## 2. Methods

**Figure 1:**
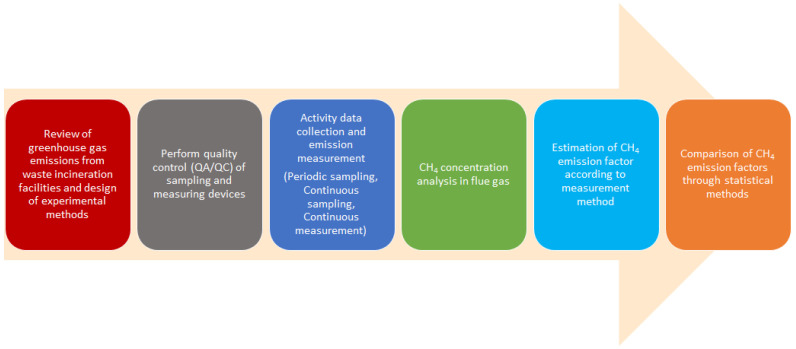
The research procedure diagram of this study.

This study proceeded according to the procedure in Figure 1 to review the methodology used to develop CH_4_ emission factors.

### 2.1. Selection of Facilities

In this study, various sampling and measurement methods were used to develop an emission factor that can reflect the representative CH_4_ emission characteristics of a facility. Three methods, including periodic sampling, continuous sampling, and continuous measurement, were used to identify their differences and compare the corresponding CH_4_ emission factor.

A household waste incineration facility was selected as the target, and the differences between the methodologies were observed by visiting the facility six times. The target facility incinerates waste using the stoker method, the most frequently used method for waste incineration in South Korea, processing 400 tons of household waste per day. The air pollution prevention facility consists of an electric dust collector facility for fine dust reduction, a bag filter, a wet scrubber facility for dust and harmful gas removal, and an SCR catalyst tower for nitrogen oxide removal. Table 1 summarizes the properties of the household waste incineration facility. 

### 2.2. Sampling and Measurement Methods

#### 2.2.1. Periodic Sampling Method

**Figure 2:**
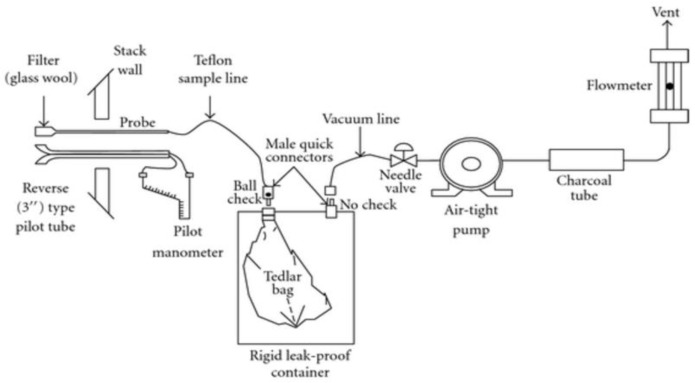
Periodic sampling using the lung sampler (EPA Method 18).

The US Environmental Protection Agency (EPA) Method 18 (US EPA, 2000), commonly used for sample collection to develop non-CO_2_ emission factors and referred to as the periodic collection method, was adopted in this study [20,21]. This method has been widely applied in South Korea for developing non-CO_2_ emission factors [22,23,24]. For the periodic collection method, a 10 L Tedlar bag (SKC, Eighty Four, PA, USA) was connected to a lung sampler (ACEN, Suwon, Korea), and a vacuum was created using the lung sampler pump to collect the sample in the bag without it passing through the pump (Figure 2). To remove the moisture contained in the emission gas, a moisture absorption vessel comprising an empty bottle and silica gel was installed in front of the sample collection device. 

#### 2.2.2. Continuous Sampling Method

The continuous collection method was devised in this study based on the method used to collect fossil carbon content samples of gas. 

**Figure 3:**
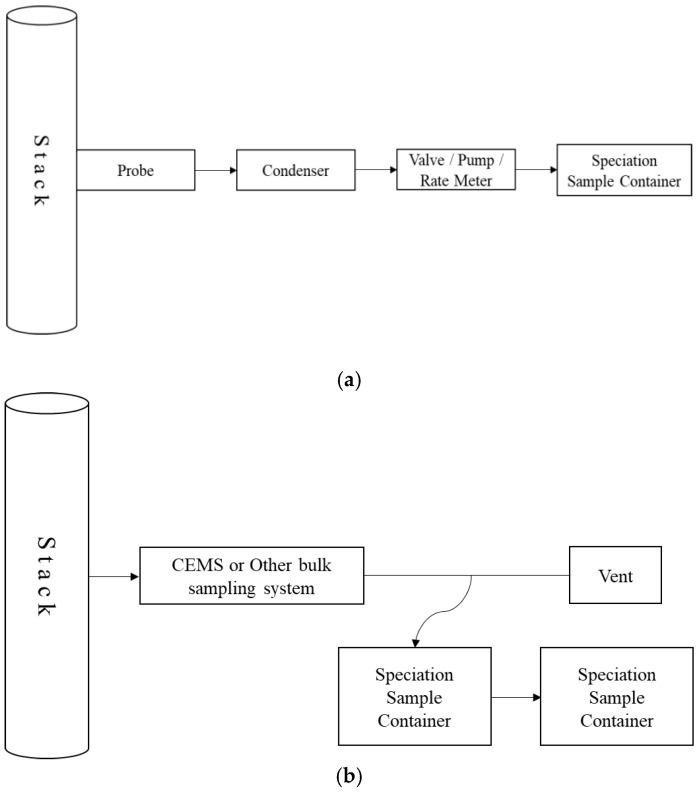
Sampling method of waste flue gas in ASTM D7459. (**a**) Sampling equipment applied to incineration facilities where CEMS is not installed; (**b**) sampling equipment applied to incineration facilities where CEMS is installed.

This method complies with the Standard Practice for Collection of Integrated Samples for the Speciation of Biomass (Biogenic) and Fossil-derived Carbon Dioxide Emitted from Stationary Emissions Sources (American Society for Testing and Materials D 7459-08, hereinafter ASTM D 7459-08) as per the mandatory reporting rule (MRR) of the EPA, which recommends the samples to be collected for at least 24 h to ensure their representativeness and proposes a sample collection diagram, as shown in Figure 3 [25,26,27].

**Figure 4:**
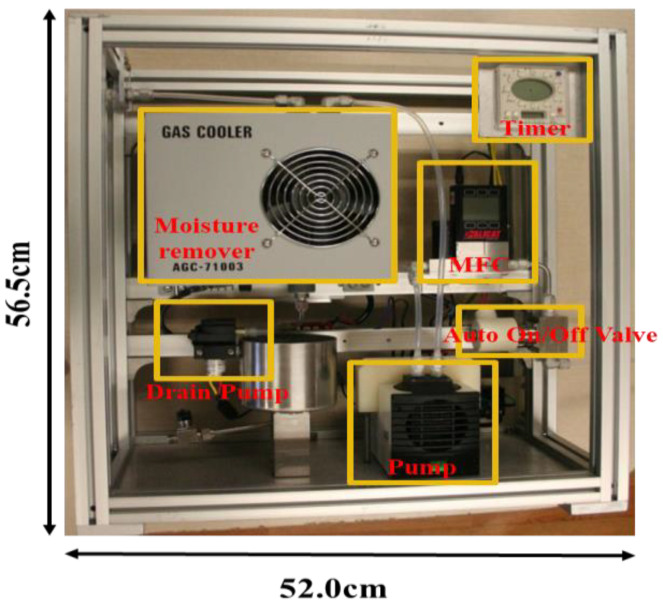
Component of continuous sampling equipment.

In this study, a sample collection device, comprising a condenser, valve, pump, rate meter, and sample container, was prepared and used to collect samples continuously (Figure 4). The continuous collector used in this study can cool the emission gas at 100 °C or higher down to 3 °C or lower through a moisture removal device (ALPHA, Gwangmyeong, Korea), and the intake flow rate can be set using a built-in electronic mass rate meter (Alicat Scientific, Tucson, AZ, USA) and pump (KNJ N86 LABOPRORT, Seoul, Korea). Further, a timer was used to allow the emission gas to be collected at specific times without any direct user intervention during the operational hours of the target facility.

The device was verified by connecting an empty Tedlar bag and another filled with 0.5 ppm of the standard gas to the rear and front ends of the continuous collection device, respectively, to confirm the difference in measured concentrations between the two Tedlar bags after the collection. Table 2 shows the results. After comparing the concentrations in the two Tedlar bags, a difference of 3% on average was observed, confirming the adequacy of the device.

#### 2.2.3. Continuous Measurement Method

Real-time measurement of CH_4_ concentration was selected as the final sampling and measurement method for the determination of the CH_4_ emission factor and is referred to as the continuous measurement method in this paper.

Although difficult to use in small spaces, the continuous measurement device provides sufficient measurement values for verifying their representativeness and other factors and has the advantage of demonstrating the emission characteristics of the target gas by acquiring continuous data.

A continuous gas chromatography flame-ionization detector (GC-FID; Synspec B.V., Groningen, The Netherlands) with a measurement period of 3 min was used for continuous CH_4_ measurement. The device can measure the total non-methane hydrocarbon (TNMHC) and CH_4_ with a measurement range of 0.01–10 ppm, respectively. A carrier gas (air, 99.999%) and H_2_ are used to measure CH_4_ (Table 3).

### 2.3. Analysis of CH_4_ Concentration Using Bag Sampling Gas

Samples collected using the periodic and continuous collection methods were analyzed using GC, where a gas sample is mixed with a moving phase (carrier gas) and passed through a stationary phase (column) to separate and quantify the various components when the sample reacts with the stationary phase. A GC-FID and a Porapack Q 80/100, 1 m stainless-steel column were used for analyzing the CH_4_ concentration. The flow rates of the carrier gas (N_2_), H_2_, and air were set at 25 mL/min, 30 mL/min, and 300 mL/min, respectively. The temperatures of the sample inlet, oven, and detector were set at 120 °C, 70 °C, and 250 °C, respectively.

**Figure 5:**
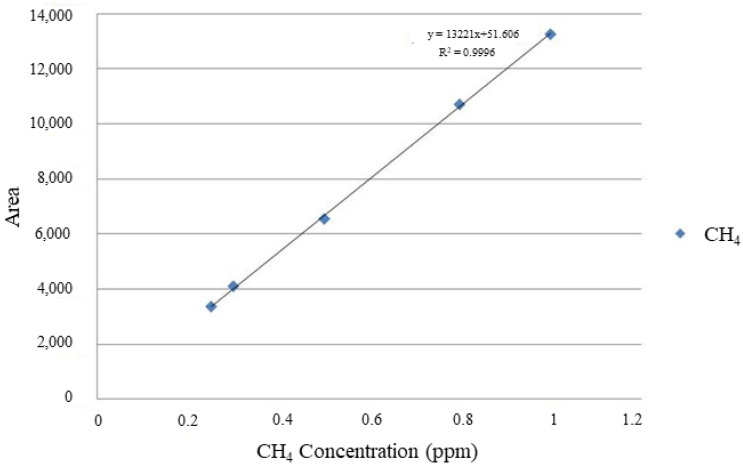
Calibration curve for CH_4_ using the standard gas.

QA/QC was performed in advance for the GC-FID used in the analysis. The calibration curve for CH_4_ analysis of gas chromatography was prepared using different concentrations of five standard gas (CH_4_) so that the concentration could be included within the range considering the concentration of CH_4_ emitted from the incineration facility. The standard gas concentrations were 0.25 ppm, 0.3 ppm, 0.5 ppm, 0.8 ppm, and 1 ppm (Rigas, Daejeon, Korea). Gas concentrations of 0.5 ppm and 1 ppm were used as the primary standard, and the secondary standard gas concentrations were prepared by diluting the primary standard gas to 0.25 ppm (diluted from 0.5 ppm), 0.3 ppm, and 0.8 ppm (diluted from 1 ppm). As shown in Figure 5, the R^2^ value of the calibration curve based on the standard sample was 0.9996, demonstrating excellent linearity.

The reproducibility of the GC-FID measurement used in this study was confirmed by repeatedly analyzing the standard gas 10 times, as shown in Table 4. The average of the analyzed measurements was expressed as a mean value. The relative standard error (RSE) was calculated to be 0.31%, which is a percentage of the standard error (SE = standard deviation/N) with respect to the mean. As the reproducibility range of the relative standard error proposed by ISO 11564:1998 is 3%, a value of 0.31% can be considered to indicate excellent reproducibility [28].

### 2.4. Estimation of CH_4_ Emission Factor at MSW Incinerator

“The Emission Calculation Methodology Based on Emission Gas Continuous Measurement Methods such as Tele-monitoring Systems” suggested by South Korea’s “Guidelines for Greenhouse Gas and Energy Target Management System” was applied to calculate the emission required for developing the CH_4_ emission factor [29]. Because the emission calculation method using the tele-monitoring system proposes a formula only for CO_2_ emission, the same was also used to determine the CH_4_ emission factor.

For calculating the daily average CH_4_ concentration used in the calculation formula, the data collected using periodic collection, continuous collection, and continuous measurement were converted to daily average concentrations. Data collected from Clean SYS, a Korean CEMS, were used as the flow rate data, and the incineration amount data were collected from the facility. 

The formula used in this study to calculate the CH_4_ emission factor is shown in Equation (1).

(1) $$Emission\;Factor_{{\mathrm{CH}}_{4}}=\left[K\times C_{{\mathrm{CH}}_{4}}\times Q_{day}\times 10^{-3}\right]/W\;$$

where $Emission\;Factor_{{\mathrm{CH}}_{4}}$ is emission factor of CH_4_ (gCH_4_/ton-waste); $C_{{\mathrm{CH}}_{4}}$ is daily average CH_4_ concentration (ppm, based on dry gas); *K* is 16/22.4 (kg/Sm^3^); *Q_day_* is accumulated flow per 1 day (kg/Sm^3^, based on dry gas); *W* is incineration amount per 1 day (ton-waste/day).

### 2.5. Statistical Test of CH_4_ Emission Factor

When inferring for a population, different statistical analysis techniques should be applied depending on the normality of the sample group [30,31]. As most statistical analyses typically assume normality of the population, non-parametric statistical methods should be used in lieu of typical test methods for abnormally distributed populations [32]. In this study, the Kolmogorov–Smirnov and Shapiro–Wilk normality tests provided by the statistical program SPSS 21 (version 21.0, IBM Corp., Armonk, NY, USA) were used to test the normality of the CH_4_ emission factors.

When a CH_4_ emission factor group demonstrates a normal population distribution, a one-way analysis of variance, a type of analysis of variance (ANOVA), is performed [33,34,35]. ANOVA can be used only when the distribution of the population corresponding to each group is normal, the variance of the population corresponding to each group is the same, and the errors within each population or between populations are independent of each other [36].

When the population is abnormally distributed and has greater than three rank measure samples, the Kruskal–Wallis test method is used. The Kruskal–Wallis test is a nonparametric test extremely similar to one-way ANOVA and allows the comparison of median and mean between groups. Sample data with abnormal distribution are treated as sequence data, and the Kruskal–Wallis test is used [37]. Although the homogeneity of variance must be satisfied as the basic assumption of one-way ANOVA, this assumption is not required for the Kruskal–Wallis test, and the test can be applied to a wider range of data.

## 3. Results

### 3.1. CH_4_ Concentrations after Sampling and Measurement

Periodic collection, continuous collection, and continuous measurement methods were used to analyze the CH_4_ concentration of the emission discharged from the household waste incineration facility. The target facility was visited six times, and the average daily CH_4_ concentrations were calculated.

Table 5 shows the CH_4_ concentrations obtained using the three sampling and measurement methods. In the case of periodic collection, nine samples were collected per visit (total 54 samples), and the measured concentration was in the range 0.385–0.681 ppm, with an overall average of 0.499 ppm.

For continuous collection, one sample was collected per visit (total eight samples), and the measured concentration ranged between 0.392 ppm and 0.676 ppm, with an overall average of 0.493 ppm.

In the case of continuous measurement, 360 CH_4_ concentration data observations were acquired per visit (total 2160 data), and the measured concentration was in the range 0.322–0.554 ppm, with an overall average of 0.437 ppm.

As a result, the periodic sampling showed the highest concentration, followed by continuous collection and continuous measurement. In the case of periodic sampling, this difference can be viewed because of not reflecting the characteristics of the night because the average value can be checked only with the value at a specific point in time. In fact, when the continuous measurement data were checked, it could be confirmed that the CH_4_ concentration at night was distributed lower than during the daytime, so it could be determined that the average concentration of the continuous measurement was relatively low. In the case of continuous collection, it was confirmed that it showed periodic sampling values between the values of periodic sampling and continuous measurement.

### 3.2. CH_4_ Emission Factor Based on Different Sampling Methods

Table 6 shows the calculation results of the CH_4_ emission factor for the three sampling and measurement methods.

The CH_4_ emission factor values obtained from the periodic collection, continuous collection, and continuous measurement methods were 0.236 gCH_4_/ton, 0.237 gCH_4_/ton, and 0.209 gCH_4_/ton, respectively. Observing the results, the continuous collection method produced the largest value, followed by periodic collection and continuous measurement. As with the differences in their concentration values, the CH_4_ emission factor exhibited a difference of less than 1% between continuous collection and periodic collection and approximately 13% for continuous measurement. Therefore, we believe that the concentration affects the emission factor. Therefore, it was confirmed that the CH_4_ emission factor was greatly affected by the concentration.

### 3.3. Statistical Comparison of Sampling Methods

We conducted a mean distribution test to statistically confirm the differences in emission factor using the statistical program SPSS 21. However, a normality test must be performed first to select the mean distribution method. As six data values (less than 2000) were used to calculate the CH_4_ emission factor, the Shapiro–Wilk test was performed. Table 7 shows the results.

The Shapiro–Wilk test results indicated that the significance probabilities of periodic collection, continuous collection, and continuous measurement were 0.386, 0.228, and 0.931, respectively. Because the significance probabilities in all three methods are greater than 0.05, the distribution can be normal. However, as the number of data values used was less than 30, the Kruskal–Wallis test was used as a nonparametric mean distribution test. The null and alternative hypotheses for the Kruskal–Wallis test were specified as follows (Table 8).

According to the test results, the null hypothesis (H0) could not be rejected because of a significance level of 0.796 (greater than 0.05). Therefore, the distribution of the CH_4_ emission factor can be statistically identical among the sampling methods, and the CH_4_ emission factors determined from the periodic collection, continuous collection, and continuous measurement methods in the household waste incineration facility exhibit no differences at the 95% confidence interval (Table 9).

## 4. Conclusions

In South Korea, recycling is the most used method for treating waste, while landfills and incineration serve as the main sources of greenhouse gas emissions. Particularly, as the amount of waste treated with incineration is increasing, relevant studies on greenhouse gas emissions and management are necessary.

Because CH_4_ and N_2_O emissions from waste incineration are calculated using emission gas measurements, the selected measurement method may significantly affect the results; therefore, it is important to examine and compare different sampling and measurement methodologies for ensuring better accuracy.

The method that has been used to develop CH_4_ emission factors in waste incineration facilities is the periodic sampling method using a lung sampler. However, since this method collects samples only at a specific point in time for a short period of time, the overall characteristics may not be reflected depending on the incineration facility (e.g., CH_4_ emission at night). As an alternative to this, there is a method to increase the sample collection time or measure the continuity. In this study, all three methods were used to calculate and compare CH_4_ emission factors.

As a result of the analysis, the average emission factor by intermittent capture was 0.201 gCH_4_/ton-waste, the average emission factor by continuous sampling was 0.199 gCH_4_/ton-waste, and the average emission factor by continuous measurement was 0.176 gCH_4_/ton-waste. There was a difference of 0.025 gCH_4_/ton-waste in the emission factor values by periodic sampling and continuous measurement, and the emission factor values for periodic sampling and continuous sampling were similar. In addition to the difference in the general average value, additional comparisons were made using a statistical method to confirm the difference in emission factors considering the distribution of the overall data. As a result of the comparison, it was confirmed that there was no statistical difference according to the three methods because the significance level was 0.796, which was greater than 0.05. Therefore, it was confirmed that all three methods can be used, which indicates that an appropriate methodology can be used in each domestic waste incineration facility considering the situation of the incineration facility.

However, because of the overall methodology review, it was confirmed that the periodic sampling still did not consider the CH_4_ emission characteristics at night when the data were reviewed. Continuous measurement had difficulties in carrying expensive equipment, difficulty in maintenance, and gas cylinders, such as hydrogen or air. On the other hand, in the case of continuous sampling, compared to continuous measurement, the cost of configuring the device is relatively low, and it has the advantage of being able to reflect the characteristics of the night by collecting samples for a longer period compared to the periodic sampling. Therefore, in this study, it is judged that the continuous collection method, which corresponds to the intermediate stage between sampling and continuous measurement, will be more representative and efficient to develop the CH_4_ emission factor.

The implications of this study are as follows:

1. The CH_4_ concentration measurement methodology was reviewed for the stoker incineration facility, and an appropriate method was suggested so that it can be referred to when developing an emission factor.
2. Statistical characteristics of CH_4_ concentration data for each CH_4_ measurement method are presented so that the relevant person in charge and researchers can refer to them when reviewing the methodology.
3. It can be used as basic data when developing the CH_4_ emission factor of the stoker incineration method among domestic waste incineration facilities by revealing the CH_4_ emission factor values and differences by various measurement methods for the stoker incineration facility.

It is expected that a review of the relevant methods for N_2_O and other incineration methods will contribute to the development of more reliable non-CO_2_ emission factors in the future.

## Figures and Tables

**Table 1:** Characteristics of the investigated MSW incinerator.

Site	Incinerator	Incineration (Ton/Day)	Air Pollution Prevention Facility	Description
A MSW incinerator	Stoker	400	Electrostatic precipitator	Particulate matter prevents
			Bag filter	Particulate matter prevents
			Wet scrubber	Hazardous gases and particulate matter prevents
			Selective catalytic reduction	Nitrogen oxide prevents

**Table 2:** Verification test of flue gas sampling equipment.

No.	Input CH_4_ Concentration(ppm) (A)	Output CH_4_ Concentration(ppm) (B)	Difference [(A − B)/A]
1	0.5	0.49	2%
2	0.5	0.49	2%
3	0.5	0.49	2%
4	0.5	0.48	4%
5	0.5	0.48	4%
Mean			3%

**Table 3:** Specifications of CH_4_ continuous measuring device.

Characteristic		Continuous Measurement Specifications
Detector		FID
Cycle time		3 min
Gas consumption		Zero air: dry and clean, methane free, 2.5 bar, 250 mL/min. Hydrogen: 3.5 bar 25 mL/min
Measuring rage	Methane	0.01–10
	TNMHC	0.01–10
Repeatability		<1% of full scale
linearity		<1% of full scale

**Table 4:** Validation of reproducibility of GC-FID.

No.	Peak Area	Concentration (ppm)
1	6634	0.50
2	6649	0.50
3	6640	0.50
4	6583	0.49
5	6488	0.49
6	6494	0.49
7	6560	0.49
8	6495	0.49
9	6593	0.49
10	6503	0.49
Mean	6564	0.49
SE (standard error)	20.62	0.00
RSE (relative standard error) (%)	0.31	0.31

**Table 5:** Comparative CH_4_ concentration by sampling method (unit: ppm).

	1	2	3	4	5	6	Mean	*n*
Periodic sampling	0.416	0.432	0.385	0.482	0.598	0.681	0.499	54
Continuous sampling	0.403	0.399	0.392	0.532	0.555	0.676	0.493	6
Continuous measurement	0.322	0.379	0.364	0.489	0.554	0.511	0.437	2160

**Table 6:** Comparative CH_4_ emission factor by sampling method (unit: gCH_4_/ton).

Periodic Sampling	Continuous Sampling	Continuous Measurement
0.236	0.237	0.209

**Table 7:** The result of normality test by CH_4_ emission factor about sampling method.

Normality Test Result		Shapiro–Wilk		
		Statistic	Degree of Freedom, DF	Sig.
Sampling method	Periodic sampling	0.902	6	0.386
	Continuous sampling	0.871	6	0.228
	Continuous measurement	0.976	6	0.931

**Table 8:** Null hypothesis by CH_4_ emission factor about sampling method.

Hypothesis Test	Content
Null hypothesis (H_0_):	the distribution of CH_4_ emission factor based on the three methods is the same.
Alternative hypothesis (H_1_):	the distribution of CH_4_ emission factor based on the three methods is different.

**Table 9:** The result of Kruskal–Wallis test by CH_4_ emission factor about sampling method.

Hypothesis Test	Null Hypothesis	Test	Sig.	Decision
Sampling method	The distribution of CH_4_ emission factor is the same across categories of sampling method	Independent samples Kruskal–Wallis test	0.796	Reject the null hypothesis

## Data Availability

Not applicable.
